# Supplementary material for: Automation, live-cell imaging, and endpoint cell viability for prostate cancer drug screens
Source: PLoS One. 2023 Oct 10;18(10):e0287126. doi: 10.1371/journal.pone.0287126 (PMC10564233; doi:10.1371/journal.pone.0287126)
Supplement: S2 File — (PDF) [file pone.0287126.s002.pdf]

Automation, live-cell imaging, and endpoint cell viability for 96-well plate drug screens

Abstract:

To streamline the identification of potentially active cancer therapeutics, here we describe a highly adaptable semi-automated approach to screen compounds simultaneously across a panel of cell lines. These protocols leverage automation to enhance robustness, reproducibility, and throughput while integrating the IncuCyte ZOOM live-cell imaging platform and the CellTiter-Glo endpoint viability assay to assess drug efficacy. The integration of both evaluative methods strategically bypasses the shortcomings of each approach individually allowing for more thorough and detailed analysis within a single drug screen. The expected output from protocol utilization includes traditional dose-response curves, IC50, area-under the curve (AUC), GR50, area-over the curve (AOC) as well as live-cell imaging to identify cell specific morphological changes, cytostatic, or cytotoxic effects of 72-hour drug treatments. This adaptable protocol can be employed across cancer model systems and represents a reproducible procedure to optimize 96-well plate cell growth conditions compatible with the integrated drug screen and simultaneously assess drug efficacy across multiple cell lines in future cancer research studies.

Before Starting:

For this protocol you will need:

- CellTiter-glo (Promega, Cat# G7572) Luminescence Viability Assay or comparable endpoint cell viability assay
- Luminometer compatible with the Promega CellTiter-Glo assay. This protocol features the Promega GloMax (Promega, Cat# GM3500) Explorer Multimode Microplate Reader.
- A liquid handler system. This protocol features the Opentrons OT-2 Robot liquid handler system.
  - OT-2 Required attachments:
    - P300 Single-Channel GEN2 Pipette
    - P300 8-Channel GEN2 Pipette
    - Opentrons 96 Tip Racks 300 µL
    - 12-Channel Reservoirs for Automation (USA Scientific, Cat# 1061-8150)
- White opaque welled, clear bottom 96-well tissue culture compatible plates. This protocol features Greiner Bio-one Cell culture microplate, 96 well, PS, F-Bottom, µClear (Sigma Aldrich,M0437-32EA)
- Sartorius IncuCyte ZOOM Live-cell analysis system or any comparable live cell imaging platform.
  - Compatible IncuCyte ZOOM Live cell analysis software.
- Sterile tissue culture hood

- Standard 8-channel p200 multi-channel pipette
- White Opaque Lab Tape
- Standard 25 mL reagent reservoirs (VWR, Cat# 89094-662)
- Standard tissue culture reagents
- Standard 96-well plate (clear)
- Standard 24-well plate (clear)

Protocol 1: 96-well plate cell growth optimization for integrated live-cell and endpoint viability drug screening assay.

To accurately integrate live-cell imaging and endpoint viability, the user must define conditions which promote cell growth, prevent over-confluence, and fall within the detectable linear range of the endpoint cell viability assay. This protocol is designed to identify the optimal cell seeding density and media conditions to be used for integrated live-cell imaging and endpoint metabolic ATP viability assays for 72-hour drug screens using 96-well plates.

*This protocol may be iteratively repeated and adjusted to obtain the user's desired 72-hour cell growth rate and final confluence.

Before starting:

- Determine the cell line to be optimized and expand cell line to ~50-60% confluence in any standard tissue t75-Flask or 15cm tissue culture dish.
- Define cell seeding density range to be tested. This protocol uses 4 seeding densities.
  Always set initial seeding density to the cell line-specific lab standard or literature recommended seeding density for 96-well plate assays. Then set a range using 50% increments (one below and two above) of initial seeding density.
  Example: If 1000 cells/well is recommended, then the range should be set based on (%50 of 1000) 500 cells/well. The initial 4 seeding increments would be 500 cells/well, 1000 cells/well, 1500 cells/well, and 2000 cells/well.
- Determine desired cell culture media conditions to be tested. This protocol 2 media conditions with varying serum concentrations. As a reference point, this protocol suggests preparing cell culture media with cell specific recommended serum concentrations and preparing cell culture with between 50-80% less serum to regulate cell growth rates.
  Example: If 10% **Fetal Bovine Serum (FBS)** is recommended, then the second concentration could be set at 5% or 2% FBS. See plate map below:

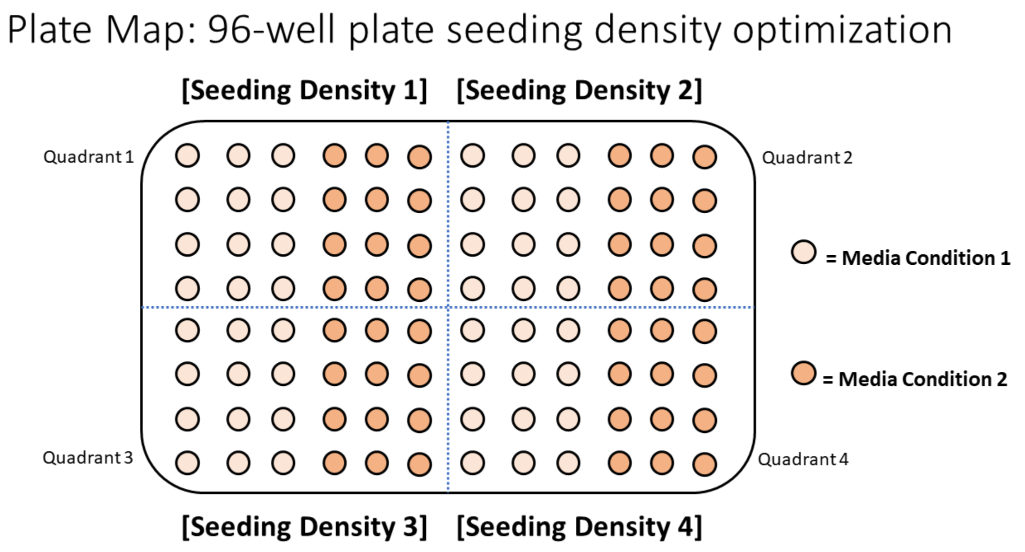

Day 1

Prepare cell lines for seeding into 96-well plates for optimization:

1. In a sterile tissue culture hood, aspirate media from t75-flask.
2. Perform a wash with 5mL Phosphate Buffered Saline (PBS) (Hyclone, Cat# SH30028.02).
3. To detach cells from tissue culture flasks, add 3mL of trypsin (Corning, Cat# 25-052-cl) directly on to the cells. Gently tilt the flask until the surface is equally covered with trypsin then place cells into tissue culture incubator for 3 minutes.
4. After incubation, gently tap the sides of the flask to insure complete detachment of adherent cells. Return to incubator for 1-minute increments if needed.
   *Use a microscope at 5-10x magnification to verify detachment.
5. Add 3mL of trypsin inhibitor (Thermofisher, Cat# R007100) (or equal volume to trypsin used) to deactivate the trypsin enzyme, collect all the cell suspension, and then transfer into a sterile 15mL conical tube.
6. Pellet cells via centrifugation at 100-300xG for 5 minutes.

   It is recommended that you prepare your media at the pre-determined optimization conditions during centrifugation. See suggestions below:

   - <u>Media condition 1</u>: General cell-specific base culture medium supplemented with antibiotics and full-serum (dependent on recommended cell line specific culture conditions). ~12mL
   - <u>Media condition 2</u>: General cell-specific base culture medium supplemented with antibiotics and 50-80% less serum than condition 1. ~12mL
   - You will also need to prepare general cell-specific base culture medium supplemented with antibiotics but SERUM FREE. ~5mL

7. After centrifugation, aspirate all supernatant while being careful to not disrupt the cell pellet.
8. Uniformly resuspend the cell pellet in 1mL of SERUM FREE media using a p1000 pipette. *Be gentle so to not shear the cells. *Then add an additional 1mL of SERUM FREE media and slowly vortex to uniformly mix
9. Using the well-mixed cell suspension, accurately count cells via your preferred hemocytometry method to a final unit of [cells/mL]

Seeding cells into 96-well plates for optimization

Cells will be seeded into 96-well plates at a volume of 200 µL/well. Thus, the concentrations that were selected should be adjusted so that there are X#ofCells/200µL of appropriate media.

<u>Example:</u> If the desired cell density range was 500 cells/well, 1000 cells/well, 1500 cells/well, and 2000 cells/well, then the adjusted concentrations should be 500 cells/200µL etc. This would result in 2500 cells/mL, 5000 cells/mL, 7500 cells/mL, and 10,000 cells/mL respectively.

10. After counting, calculate the volume of cells suspended in SERUM FREE media needed to prepare 12 mL at the adjusted seeding density concentration range previously determined.

Note: In this protocol, each seeding density and seeding density are tested in replicates of 12. Thus, at 200 µL/well, 2.4 mL of each media condition will be the minimum required volume per seeding density (See plate map above). To find the requisite volume of cell suspension, use the formula: $V_1 = \frac{C_2 V_2}{C_1}$ where V_1= Desired volume of cell suspension, C_1= Concentration [cells/mL] of cell suspension, C_2=Desired final concentration [cells/mL] of new cell preparation, & V_2= Desired final volume of new cell preparation

11. Using the two media conditions prepared between steps 6 & 7, prepare 3 mL of each seeding density/ media condition combination in separate sterile 15 mL conical tubes.

Example table:

Cell Line	Seeding Density	Media Condition	Final Volume
**Cell X**	500 cells/well	Condition 1	3mL
**Cell X**	1000 cells/well	Condition 1	3mL
**Cell X**	1500 cells/well	Condition 1	3mL
**Cell X**	2000 cells/well	Condition 1	3mL
**Cell X**	500 cells/well	Condition 2	3mL
**Cell X**	1000 cells/well	Condition 2	3mL
**Cell X**	1500 cells/well	Condition 2	3mL
**Cell X**	2000 cells/well	Condition 2	3mL

12. One at a time, gently vortex each tube and transfer into a standard sterile standard 25 mL reagent reservoir (VWR, Cat# 89094-662). Then using an 8-channel p200 multi-channel pipette, transfer the cell suspensions into a cell culture 96-well microplate with white opaque walls and a clear bottom (Sigma Aldrich, M0437-32E) at their proper locations as specified by the above plate map.

*Note: These types of plates are required for the execution of the integrated live-cell and endpoint viability drug screen as live cell imaging requires clear bottoms for imaging and endpoint viability assays work best with wells isolated by opaque walls.

13. Place 96-well plate into standard tissue culture incubator overnight.

Day 2

Verify cell attachment to plates and begin IncuCyte ZOOM live-cell imaging:

1. Observe cells with a microscope at 10x magnification to verify that cells are settled and adhered to the inner surface of the 96-well plate. Verify that there is a reasonable difference in expected seeding densities.

*Ask the question: "Do cells seeded at 2000 cells/well appear ~4 times more confluent than cells seeded at 500 cells/well?"

2. At this stage, the plate can be transferred to the preferred live-cell imaging platform. This protocol uses the IncuCyte Zoom platform. The plate will remain in the imaging platform for 72-hours (the duration of the drug screens in the integrated live-cell and endpoint viability drug screen protocol).

The instructions below apply exclusively to the IncuCyte Zoom platform which this protocol features (Note: basic user instructions for the IncuCyte Zoom can be found in publicly available online videos).

Begin IncuCyte Zoom set up:

- Open IncuCyte Zoom software on computer desktop.
- Connect to device.
- Under the "Task List" panel on the left-hand side, select "Schedule Scans".
- Click one of the "Empty" slots on the live representative plate map for the hardware then click "Add Vessel".
- Once prompted, search from the vessel (96-well plate) by catalog number. This protocol features the Greiner bio one cell culture microplate (#655098). Once selected proceed to setting up experimental parameters:
- On the bottom panel on the left-hand side, select "Edit Scan Pattern" and select all wells and set the scan pattern to 4 images/well. Save this scan pattern.
- In the Channel Selection section in the center, click "Phase" (no colored acquisition channel is need for this protocol).
- In the "Scan Mode" section in on the top-right side, toggle to the scan pattern that was previously created and saved.
- In the "Analysis Job Setup" section on the right-hand side, toggle the "Job Type" and select "Basic Analyzer." Toggle the "Processing Definition" tab and select a pre-determined processing definition with masks optimized for your specific cell line. (If this hasn't been created, the "DEMO Phase" processing definition can be used.
- Click the "Properties" tab and label the plate as desired.

- Click “Apply” on the bottom right corner to save changes and register the plate to the IncuCyte Zoom hardware.

Aside: Set the scan intervals based on desired timepoints. This protocol recommends a scan every 4 hours.

3. Transfer the 96-well plate into the same IncuCyte Zoom slot selected during the software setup and begin real-time image capture.

*THIS PORTION OF THE PROTOCOL WILL LAST FOR ~72 HOURS. THE LIVE-CELL IMAGING PROCESS SHOULD NOT REQUIRE ANY ADDITIONAL ADJUSTMENTS DURING THIS TIME; HOWEVER, THE USER CAN CHECK IMAGES DAILY TO TRACK PROGRESS AND VERIFY IMAGE FOCUS AND SCAN QUALITY. *

Day 5

End live-cell imaging procedure and perform CellTiter-glo endpoint viability assay.

Before Starting:

Required Materials:

- White opaque tape
- Standard 8-channel p200 multi-channel pipette
- Standard 25 mL reagent reservoirs (VWR, Cat# 89094-662)
- CellTiter-glo (Promega, Cat# G7572) Luminescence Viability Assay or comparable endpoint cell viability assay
- Luminometer compatible with the Promega CellTiter-Glo assay. This protocol features the Promega GloMax (Promega, Cat# GM3500) Explorer Multimode Microplate Reader.

*****Pre-mixed CellTiter-glo reagent is stored at -80°C (pre-aliquot 10mL into 15mL conical tubes) so begin thawing (in a dark environment) to room temperature upon arrival to lab. It will take approximately 3-4 hours to equilibrate to room temperature.

1. After 72-hours have passed, end the experiment on the live-cell imagining platform software then remove the 96-well plate and allow and allow it to equilibrate to room temperature ~ 20 minutes. (This can be done in a non-sterile environment such as a bench top or isolated drawer).

*Use the following instructions to end the experiment on the IncuCyte ZOOM software.

End IncuCyte Zoom Experiment:

- Open IncuCyte Zoom software on computer desktop.
- Connect to device.
- Under the “Task List” panel on the left-hand side, select “Schedule Scans”.
- Click on the slot housing the plate being tested on the live representative plate map for the hardware then click “Remove Vessel”.

- Click “Apply” on the bottom right corner to save changes.
- Manually remove the corresponding 96-well plate to the IncuCyte Zoom hardware.

2. Cover the bottom of the 96-well plate with white opaque lab tape.
   *Note: Cover the bottom of each plate with opaque white tape (as recommended by Promega) to prevent any luminescence decrease or “cross talk” when performing the CellTiter-glo assay as the luminometer reads the each well from the top of the plate.

   See example below:

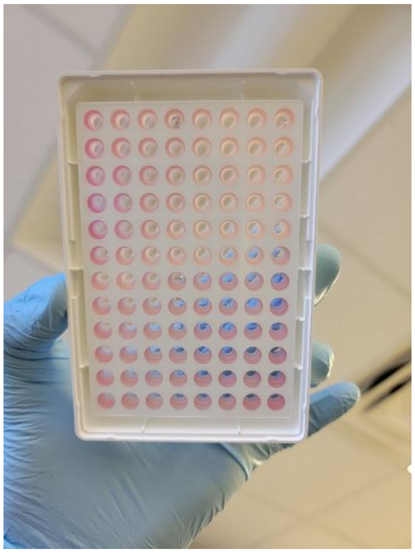

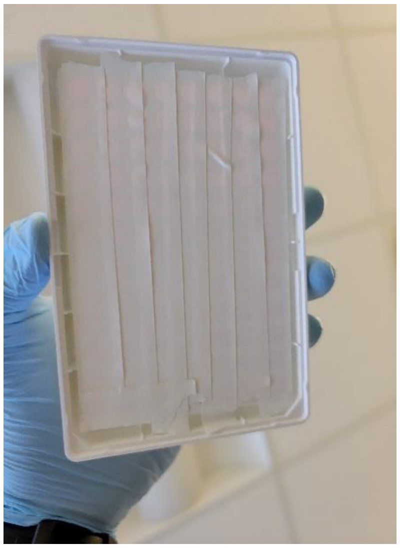

3. Transfer 10mL (per plate) of the thawed CellTiter-Glo reaction reagent into a standard 25 mL reagent reservoir.
4. Use an 8-channel p200 multi-channel pipette (or comparable multi-channel pipette) to transfer 100uL of CellTiter-Glo reaction reagent into each well of the 96-well plate.
5. Leave the plate cover off and transfer into a luminometer compatible with CellTiter-glo. Be mindful of the plate orientation and alignment to insure proper placement into the device.
   See example below:

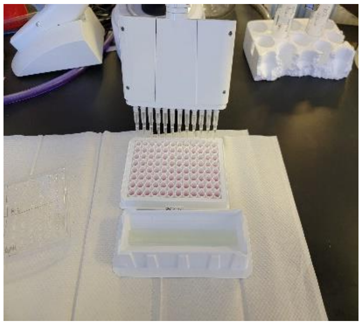

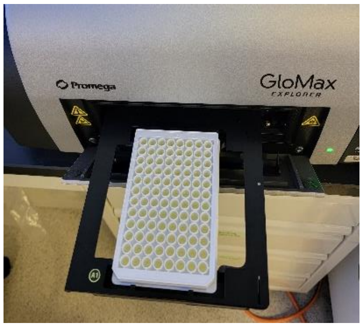

* This protocol features the Promega GloMax (Promega, Cat# GM3500) Explorer Multimode Microplate Reader.

Recommended parameters for the Luminometer are as follows:

i. Shake in an orbital shaker for 2 minutes at 500 cycles/minute with a 1mm shaking diameter (cell lysis).
ii. Incubate for 10 minutes in a dark environment (reaction).
iii. Read luminescence of each well at an integration of 400ms (data acquisition)

*It will take ~13 minutes to read each plate

6. Export data and remove plate from luminometer.

This protocol is designed to identify optimized seeding conditions for a single cell line so that it is compatible for sequential live-cell imaging and endpoint viability assays in a 72-hour drug screen. Successfully optimized seeding densities and conditions meet the following basic criteria:

I. Live-cell imaging displays healthy cells which are growing throughout 72-hours.
II. Cells aren't overconfluent at 72-hour endpoint. (We recommend a final confluence ≤ 60%, however, some slow growing cell lines may need to be seeded more densely and, in these cases, avoid conditions where cells are growing in clumps at experimental endpoint).
III. The range of seeding densities (at each condition) fall within the detectable linear range of the endpoint viability assay.

The user may find a range of options that fit these criteria. In these cases, it is to the user's discretion to select the conditions that align best with their study objective.

Tips for data analysis:

- **Track confluence throughout live-cell imaging steps- at endpoint export confluence data for all wells as an average of each of the images. This will allow you to calculate growth rate as well as final average endpoint confluence.**
- **To determine if seeding densities are within the detectable linear range of the endpoint viability assay, Create an X-Y scatterplot where raw luminescence values averaged for each replicate is graphed at each seeding density. Perform a linear regression or linear curve fit to calculate the R^2 (a value which denotes how well the data "fits" the linear curve). Note: R^2 values range from 0-1 where ≥ 0.9 is considered a good fit.**
- **If users are planning to optimize multiple cell lines for comparative drug screens, then it is recommended to select conditions which allow the most similarities across cell lines. I.e. if one cell line is only within an optimized range when 2% FBS is used but another is in an optimized ranged at either 2% or 5% FBS, proceed with both cell lines using 2% FBS to limit variables.**

Protocol 2: 96-well plate OT-2 liquid handler integrated live-cell and endpoint viability drug activity screen.

To overcome the individual shortcomings of live-cell imaging and endpoint viability, it is recommended to integrate both cell viability assessment methods in a single drug screen. This protocol is designed to evaluate drug efficacy simultaneously robustly and reproducibly across multiple cell lines through the integration of automation, live-cell imaging, and an endpoint metabolic ATP viability assay.

*It is recommended that this protocol be used after defining optimized assay-specific growth conditions through the application of the protocol "96-well plate cell growth optimization for integrated live-cell and endpoint viability drug screening assay" for each tested cell line.

***Note this protocol can be adapted to accommodate user preferences, but the Opentrons OT-2 procedures included were designed to test 8 cell lines simultaneously.**

Before starting:

- Select the drug to be tested and determine the max concentration to be tested.
    - Starting with the defined max concentration, this protocol tests a single drug at 6 concentrations (including a vehicle control) at 1:3 serial dilutions.
- Determine the cell line models which will be tested. Expand cell lines to ~50-60% confluence in any standard tissue t75-Flask or 15cm tissue culture dish.
- Determine cell-specific seeding density and media condition to be tested.

Day 1

Cell Seeding for 96-well plate drug screening assay.

Before Starting:

Required Materials:

- A liquid handler system. This protocol features the Opentrons OT-2 Robot liquid handler system
    - OT-2 Required attachments:
    - P300 8-Channel GEN2 Pipette
    - Opentrons 96 Tip Racks 300 µL
    - 12-Channel Reservoirs for Automation (USA Scientific, Cat# 1061-8150)
- White opaque welled, clear bottom 96-well tissue culture compatible plates. This protocol features Greiner Bio-one Cell culture microplate, 96 well, PS, F-Bottom, µClear (Sigma Aldrich,M0437-32EA)
- Standard tissue culture reagents (Cell-specific)
- Sterile tissue culture hood

Prepare cell lines for seeding into 96-well plates via Opentrons OT-2 liquid handler:

1. In a sterile tissue culture hood, aspirate media from t75-flask. ***Note: It is good practice to always work with one cell line in the sterile tissue culture hood at any given time.**
2. Perform a wash with 5mL Phosphate Buffered Saline (PBS) (Hyclone, Cat# SH30028.02).

3. To detach cells from tissue culture flasks, add 3mL of trypsin (Corning, Cat# 25-052-cl) directly on to the cells. Gently tilt the flask until the surface is equally covered with trypsin then place cells into tissue culture incubator for 3 minutes.
4. After incubation, gently tap the sides of the flask to insure complete detachment of adherent cells. Return to incubator for 1-minute increments if needed.
   *Use a microscope at 5-10x magnification to verify detachment.
5. Add 3mL of trypsin inhibitor (Thermofisher, Cat# R007100) (or equal volume to trypsin used) to deactivate the trypsin enzyme, collect all the cell suspension, and then transfer into a sterile 15mL conical tube.
6. Pellet cells via centrifugation at 100-300xG for 5 minutes.
7. After centrifugation, aspirate all supernatant while being careful to not disrupt the cell pellet.
8. Uniformly resuspend the cell pellet in 1mL of cell-specific culture media using a p1000 pipette. *Be gentle so to not shear the cells. *Then add an additional 1mL of cell-specific culture media and slowly vortex to uniformly mix
9. Using the well-mixed cell suspension, accurately count cells via your preferred hemocytometry method to a final unit of [cells/mL]
10. After counting, calculate the volume of cells suspended in cell-specific culture media needed to prepare 10 mL cells at the seeding density concentration range previously determined.

*For each of the 8 cell lines, 6 drug concentrations will be tested in quadruplicate. Each cell line will be seeded into a designated quadrant of a single white opaque walled, clear bottomed 96-well plate- thus TWO 96-well plates are required to test 8 cell lines. The seeding volume is 200 μL per well.

Note: If OT-2 or any other comparable liquid handler is not being used, then proceed to seed each plate according to the quadrants shown below:

11. Transfer 10mL of each cell suspension to columns 1-8 of a 12-channel reservoir. See schematic below.

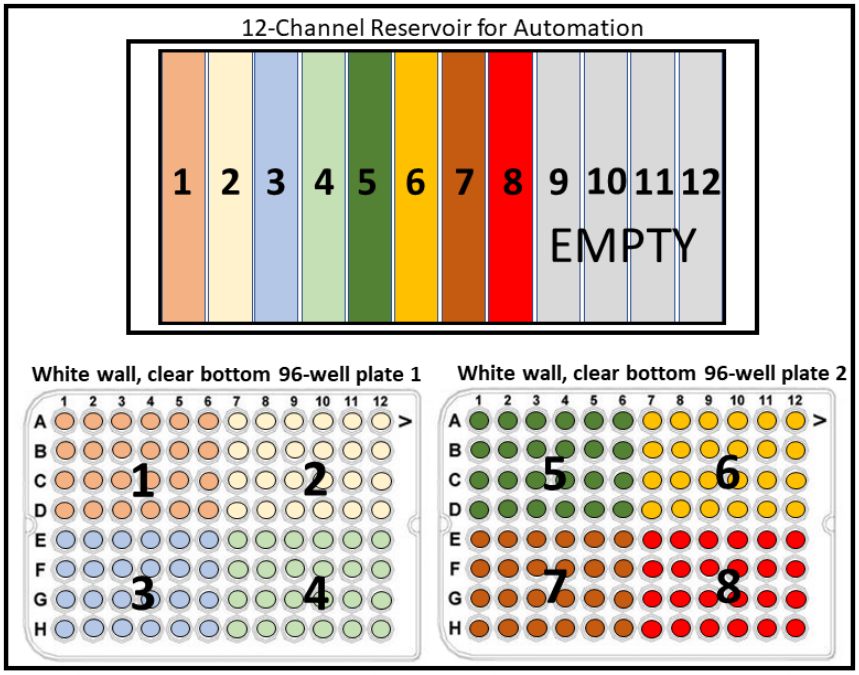

Preparing for cell seeding via Opentrons OT-2 Liquid handler

- This protocol features the Opentrons OT-2 liquid handler for cell seeding, drug dilution, and drug delivery to cells.
- For cell seeding, the following OT-2 protocol can be used to, resuspend/mix cell suspension, and transport them to their respective quadrants as defined in the schematic above.

IMPORTANT NOTE:

To test 4 cell lines per 96-well plate at 6 different concentrations, cell lines must be seeded separately within a specified quadrant of the plate. The OT-2 liquid handler system utilizes an 8-channel pipettor attachment which is designed to pick up pipette tips for all 8-channels. The Opentrons OT-2 pipettor cannot be adjusted to only pick up 4 pipette tips at a time. For this reason, before seeding users must rearrange tip rack patterns in a sterile environment.

Attached File: “Cell Seeding 8 Cell Lines.json”
Duration: ~12 minutes

Instructions for tip arrangement:

- Alternate between a full row of tips (for mixing in the 12-channel reservoir) in one row and then 4 tips for the corresponding quadrant where the cells will seeded. This protocol seeds the cells in the upper quadrants first, and then returns to the cells in the lower quadrants from left to right. Two tip racks will be needed for this protocol. See image below:

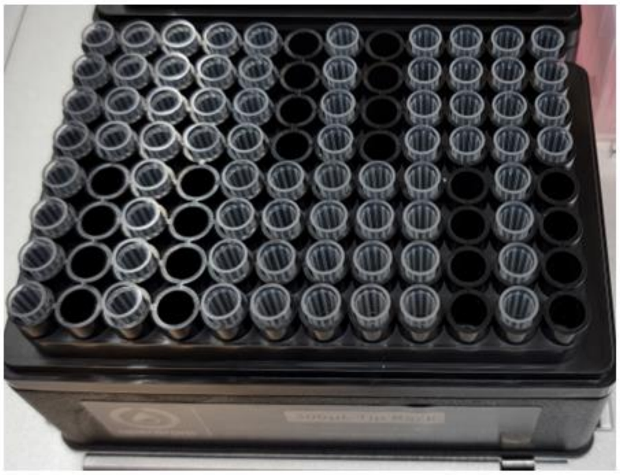

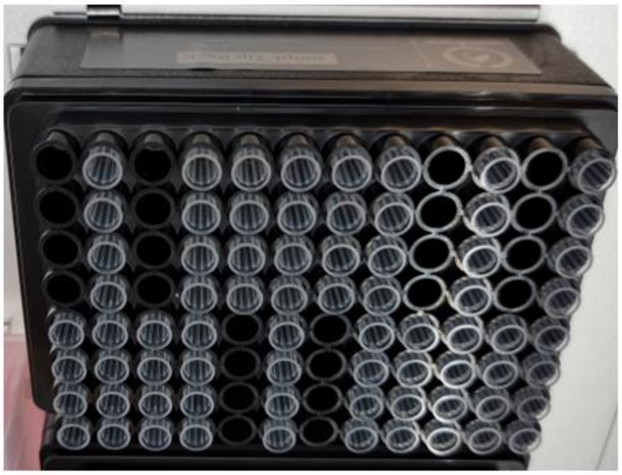

Corresponding starting deck state compatible with OT-2 seeding protocol:

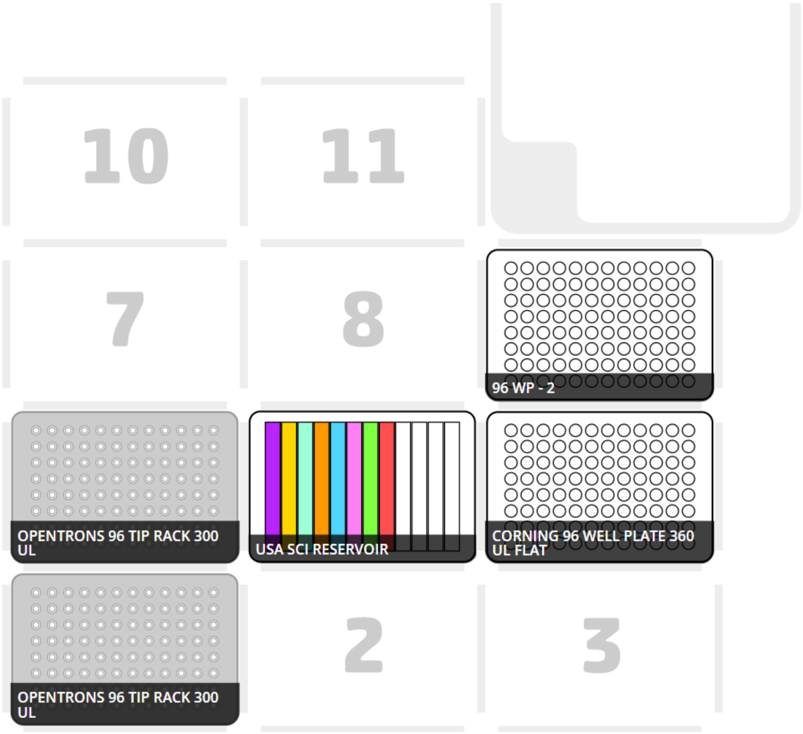

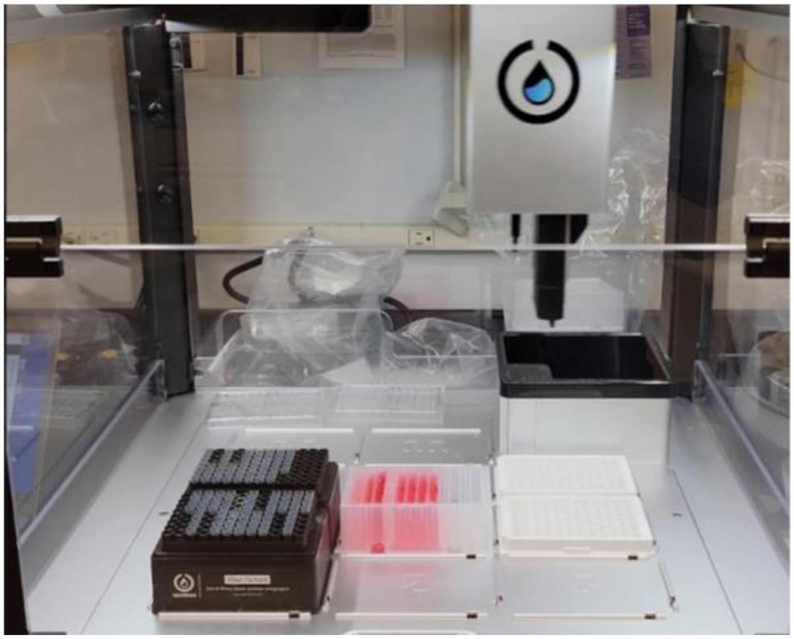

12. Transfer loaded 12-channel reservoir, pre-arranged tip racks, and two empty white walled/clear bottomed 96-well plates into the OT-2 deck and begin OT-2 procedure “Cell Seeding 8 Cell Lines.json”.
13. Place 96-well plates into standard tissue culture incubator overnight.

Day 2

Drug preparation, drug dilution/delivery with OT-2 liquid handler, and IncuCyte ZOOM set up.

Before Starting:

Required Materials:

- A liquid handler system. This protocol features the Opentrons OT-2 robot liquid handler system.
  - OT-2 Required attachments:
    - P300 Single-Channel GEN2 Pipette
    - P300 8-Channel GEN2 Pipette
    - Opentrons 96 Tip Racks 300 µL
- Sartorius IncuCyte ZOOM Live-cell analysis system or any comparable live cell imaging platform.
  - Compatible IncuCyte ZOOM Live cell analysis software.
- Standard 96-well plate (clear)
- Standard 24-well plate (clear)
-

Prepare drug dilutions.

1. Observe cells with a microscope at 10x magnification to verify that cells are settled and adhered to the inner surface of the 96-well plate.
2. Prepare drug at pre-determined max concentration into pre-determined optimized media conditions. For this protocol, each cell line (in the optimized media conditions) will require 2.5µL of drug prepared at the pre-determined max concentration as well as 2.5µL of vehicle treated media at the same percentage of the max drug concentration.

Example: If you are working with DrugX at a stock concentration of 50mM and desire a max concentration of 30µM in 2.5 mL of media use the formula: $V_1 = \frac{C_2 V_2}{C_1}$ where V_1= Desired volume of DrugX at stock concentration to be added into optimized media conditions, C_1= Concentration [µM] of DrugX at stock concentration, C_2=Desired final concentration of DrugX (30µM), & V_2= Desired final volume of new cell preparation (2.5mL). For the vehicle preparation, add equal volume as V_1 of vehicle into media with optimized conditions.

*For the OT-2 protocol drug dilutions are prepared in 24-well plates according to the plate map below (continuing with the example above):

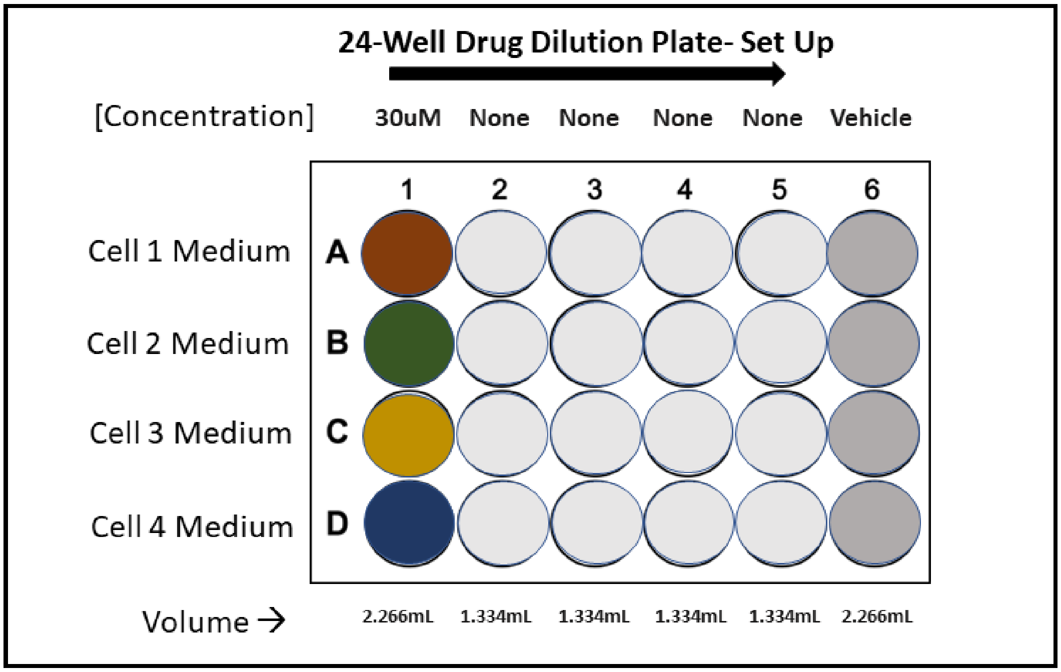

3. In a sterile 24-well plate transfer 2.266 mL of DrugX at max concentration in column 1 for each cell line using the cell-specific media conditions. Similarly, transfer 2.266 mL of vehicle treated media into column 6. In the center columns 2-5, add 1.334 mL of untreated media at optimized conditions for the corresponding cell lines (See plate map above). There should be one 24-well plate per white opaque 96-well plate (seeded the previous day).

IMPORTANT NOTE:

To create 1:3 serial dilutions, this protocol uses the Opentrons OT-2 liquid handler. The OT-2 protocol uses the P300 Single-Channel GEN2 Pipette to perform the dilutions and then transfers the drug into a template standard clear 96-well plate. There is a pause in the protocol at this step so that the first 96-well opaque white, clear bottom can be added to the OT-2. Afterwards, on the first column, the P300 8-Channel GEN2 Pipettor slowly (to not disrupt adherent cells) removes 200 µL of media that cells were seeded in on day 1 then replaced with 100 µL of drug from the template 96-well plate first column. This is repeated until the plate is treated fully.

Drug dilution and transfer to template clear 96-well plate schematic:

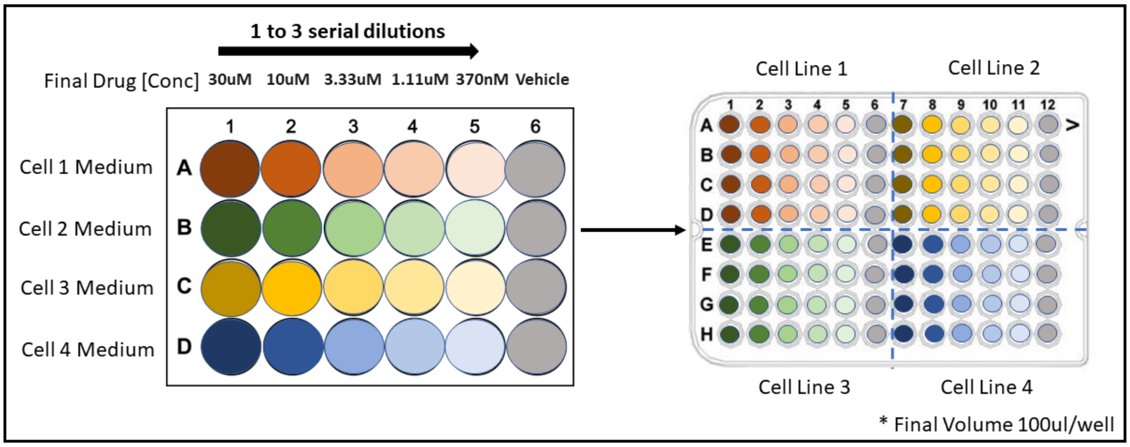

Attached File: “Drug Dilution and Transfer.json”
Duration: ~65 minutes

Corresponding starting deck state compatible with OT-2 seeding protocol:

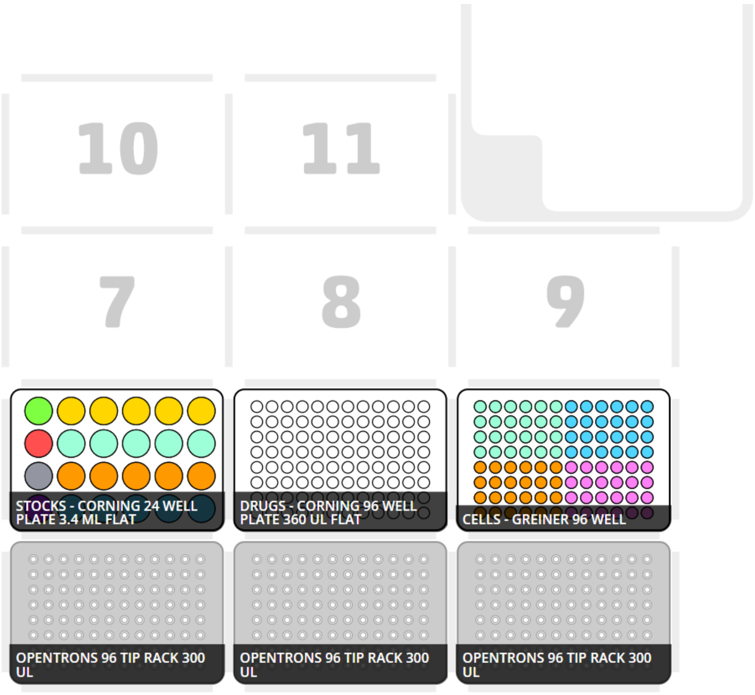

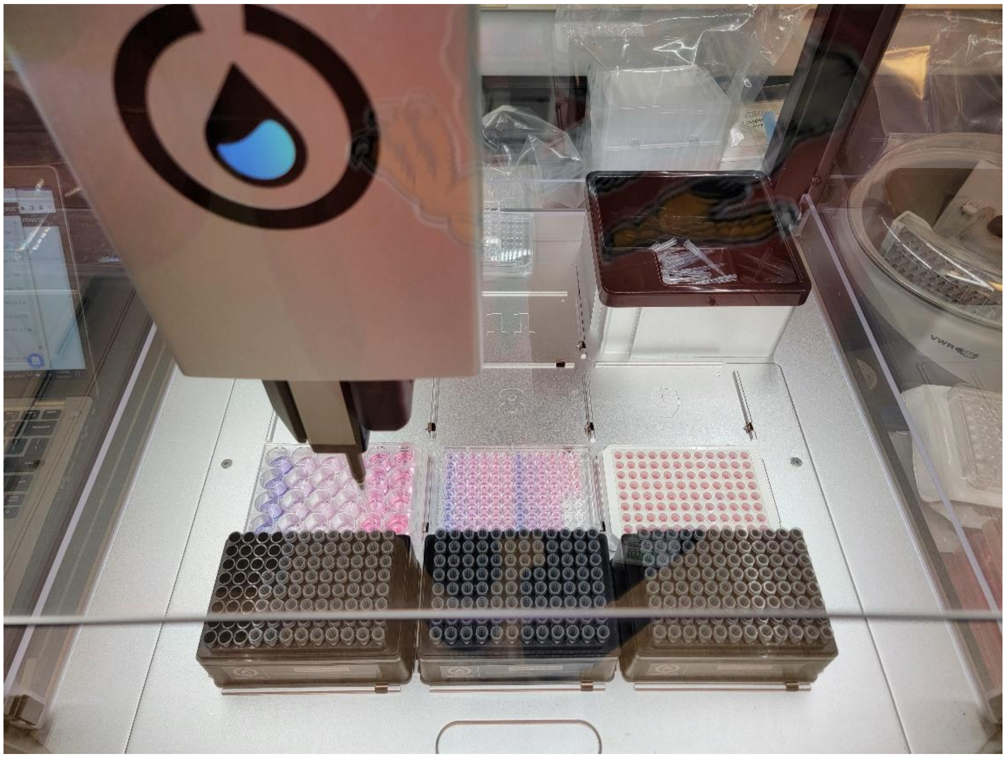

4. Transfer loaded 24-well plate, full tip racks, an empty standard clear 96-well plates into the OT-2 deck and begin OT-2 procedure “Drug Dilution and Transfer.json”.

5. After drug dilution and transfer into the template standard clear 96-well plate (~43 minutes), the protocol pauses to allow the user to add the first 96-well plate at this step. Add plate then resume protocol.
6. After the first plate is completed, return treated white 96-well plate into an incubator, and repeat the drug dilution and treatment procedure with the second plate (~65 mins).
7. Afterwards, second plate into the incubator and proceed to the next step.

Verify cell attachment to plates after drug treatments and begin IncuCyte ZOOM live-cell imaging:

8. At this stage, the plate can be transferred to the preferred live-cell imaging platform. This protocol uses the IncuCyte Zoom platform. The plate will remain in the imaging platform for 72-hours (the duration of the drug screens in the integrated live-cell and endpoint viability drug screen protocol).

The instructions below apply exclusively to the IncuCyte Zoom platform which this protocol features (Note: basic user instructions for the IncuCyte Zoom can be found in publicly available online videos).

Begin IncuCyte Zoom set up:

a. Open IncuCyte Zoom software on computer desktop.
b. Connect to device.
c. Under the “Task List” panel on the left-hand side, select “Schedule Scans”.
d. Click one of the “Empty” slots on the live representative plate map for the hardware then click “Add Vessel”.
e. Once prompted, search from the vessel (96-well plate) by catalog number. This protocol features the Greiner bio one cell culture microplate (#655098). Once selected proceed to setting up experimental parameters:
f. On the bottom panel on the left-hand side, select “Edit Scan Pattern” and select all wells and set the scan pattern to 4 images/well. Save this scan pattern.
g. In the Channel Selection section in the center, click “Phase” (no colored acquisition channel is need for this protocol).
h. In the “Scan Mode” section in on the top-right side, toggle to the scan pattern that was previously created and saved.
i. In the “Analysis Job Setup” section on the right-hand side, toggle the “Job Type” and select “Basic Analyzer.” Toggle the “Processing Definition” tab and select a pre-determined processing definition with masks optimized for your specific cell line. (If this hasn’t been created, the “DEMO Phase” processing definition can be used.
j. Click the “Properties” tab and label the plate as desired.
k. Click “Apply” on the bottom right corner to save changes and register the plate to the IncuCyte Zoom hardware.

Aside: Set the scan intervals based on desired timepoints. This protocol recommends a scan every 4 hours.

9. Transfer the 96-well plates into the same IncuCyte Zoom slot selected during the software setup and begin real-time image capture.

*THIS PORTION OF THE PROTOCOL WILL LAST FOR ~72 HOURS. THE LIVE-CELL IMAGING PROCESS SHOULD NOT REQUIRE ANY ADDITIONAL ADJUSTMENTS DURING THIS TIME; HOWEVER, THE USER CAN CHECK IMAGES DAILY TO TRACK PROGRESS AND VERIFY IMAGE FOCUS AND SCAN QUALITY. *

Day 5

End live-cell imaging procedure and perform CellTiter-glo endpoint viability assay.

Before Starting:

Required Materials:

- White opaque tape
- Standard 8-channel p200 multi-channel pipette
- Standard 25 mL reagent reservoirs (VWR, Cat# 89094-662)
- CellTiter-glo (Promega, Cat# G7572) Luminescence Viability Assay or comparable endpoint cell viability assay
- Luminometer compatible with the Promega CellTiter-Glo assay. This protocol features the Promega GloMax (Promega, Cat# GM3500) Explorer Multimode Microplate Reader.

*****Pre-mixed CellTiter-glo reagent is stored at -80°C (pre-aliquot 10mL into 15mL conical tubes) so begin thawing (in a dark environment) to room temperature upon arrival to lab. It will take approximately 3-4 hours to equilibrate to room temperature.

1. After 72-hours have passed, end the experiment on the live-cell imagining platform software then remove the 96-well plate and allow and allow it to equilibrate to room temperature ~ 20 minutes. (This can be done in a non-sterile environment such as a bench top or isolated drawer).

*Use the following instructions to end the experiment on the IncuCyte ZOOM software.

End IncuCyte Zoom Experiment:

a. Open IncuCyte Zoom software on computer desktop.
b. Connect to device.
c. Under the “Task List” panel on the left-hand side, select “Schedule Scans”.
d. Click on the slot housing the plate being tested on the live representative plate map for the hardware then click “Remove Vessel”.
e. Click “Apply” on the bottom right corner to save changes.
f. Manually remove the corresponding 96-well plate to the IncuCyte Zoom hardware.

2. Cover the bottom of the 96-well plate with white opaque lab tape.
   *Note: Cover the bottom of each plate with opaque white tape (as recommended by Promega) to prevent any luminescence decrease or “cross talk” when performing the CellTiter-glo assay as the luminometer reads the each well from the top of the plate.

   See example below:

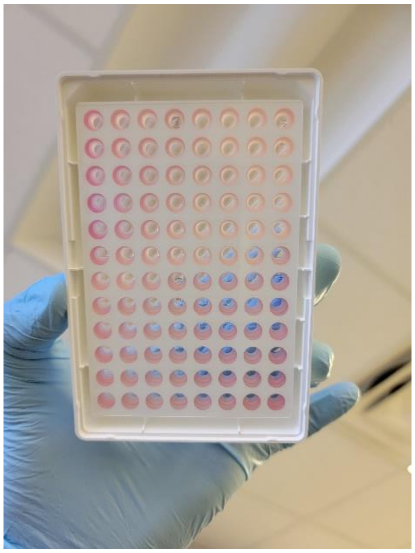
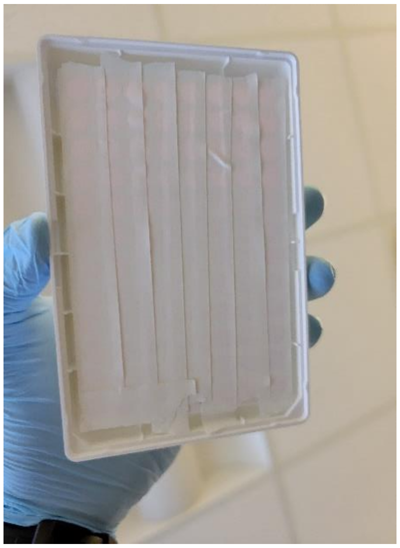

3. Transfer 10mL (per plate) of the thawed CellTiter-Glo reaction reagent into a standard 25 mL reagent reservoir.
4. Use an 8-channel p200 multi-channel pipette (or comparable multi-channel pipette) to transfer 100uL of CellTiter-Glo reaction reagent into each well of the 96-well plate.
5. Leave the plate cover off and transfer into a luminometer compatible with CellTiter-glo. Be mindful of the plate orientation and alignment to insure proper placement into the device.
   See example below:

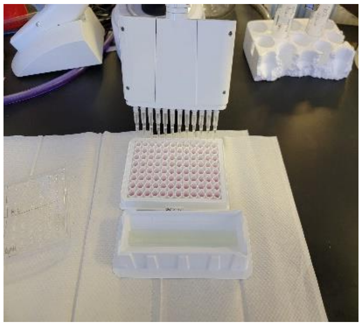
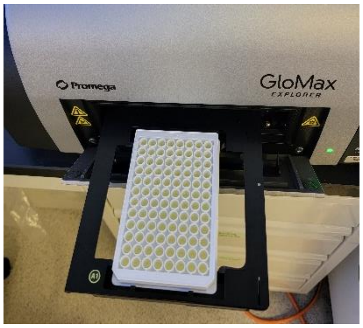

* This protocol features the Promega GloMax (Promega, Cat# GM3500) Explorer Multimode Microplate Reader.

Recommended parameters for the Luminometer are as follows:

iv. Shake in an orbital shaker for 2 minutes at 500 cycles/minute with a 1mm shaking diameter (cell lysis).
v. Incubate for 10 minutes in a dark environment (reaction).
vi. Read luminescence of each well at an integration of 400ms (data acquisition)

*It will take ~13 minutes to read each plate

6. Export data and remove plate from luminometer.

This protocol is designed to simultaneously screen a single compound across a panel of cell lines in a semi-automated procedure integrating sequential live-cell imaging and endpoint viability assays in a 72-hour drug screen. Upon successful execution of this protocol the user will have access to the following raw data:

I. Live-cell imaging to display morphological changes and response to drug treatment at 6 concentrations per cell line over a 72-hour time span.
II. Endpoint cell metabolism-based viability data curves generating dose response curves for each cell line.
III. Untreated cell growth rates and cell viability drug response data which allows for the calculation of metrics such as IC50, AUC (area under the curve), GR50, and AOC (area over the curve).
