# Supplementary figures and images for: Automation, live-cell imaging, and endpoint cell viability for prostate cancer drug screens
Source: PLoS One. 2023 Oct 10;18(10):e0287126. doi: 10.1371/journal.pone.0287126 (PMC10564233; doi:10.1371/journal.pone.0287126)

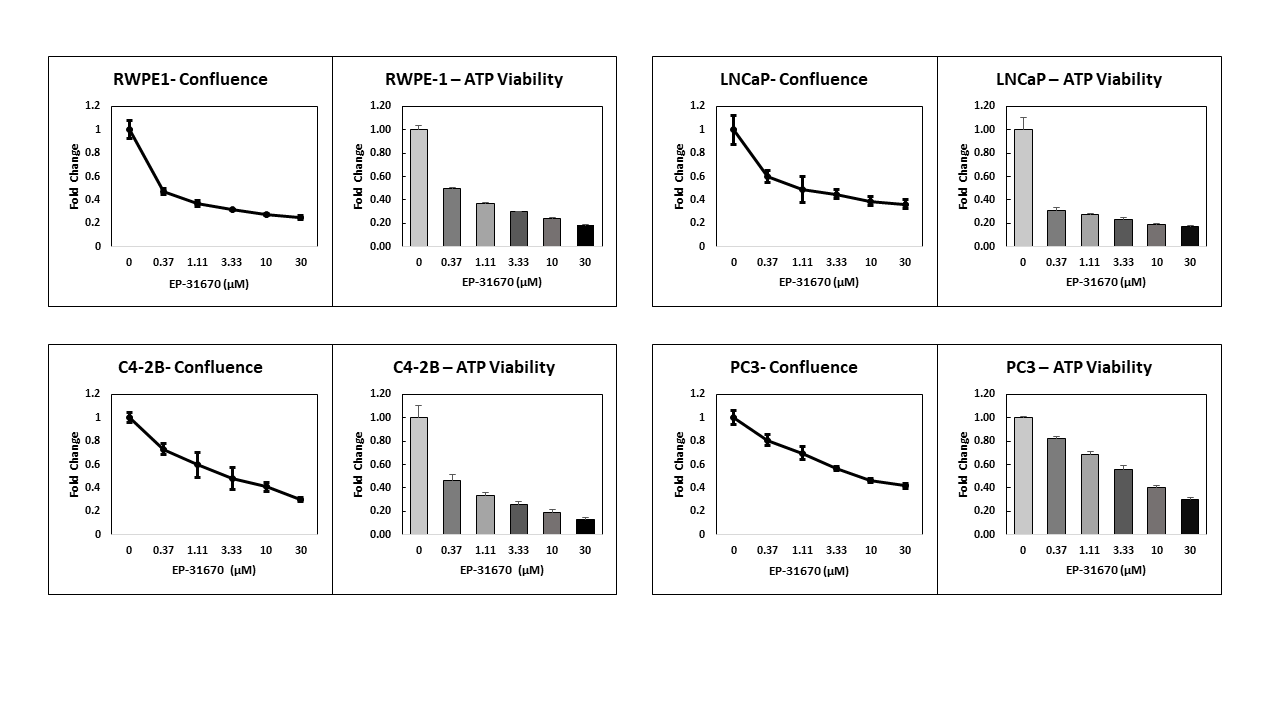

Supplement: S1 Fig — This figure was generated using data from the same experiment described in Fig 6. Non-tumorigenic (NT) prostate cells RWPE-1, androgen-dependent prostate cancer (ADPC) LNCaP, castration-resistant prostate cancer (CRPC) C4-2B, and AR-null CRPC PC3 cells were seeded in a 96-well plate at optimized conditions and treated for 72-hours (as described in Fig 6). These representative curves for each cell line were generated to demonstrate consistency in assessing drug response via endpoint confluence with the IncuCyte ZOOM and endpoint viability using CellTiter-Glo ATP viability data at 72-hrs for the tested cell lines. Fold change growth normalized to the lowest drug concentration 0 (vehicle) for each concentration was calculated ± SD (quadruplicate replicates). (TIF) [file pone.0287126.s003.tif]

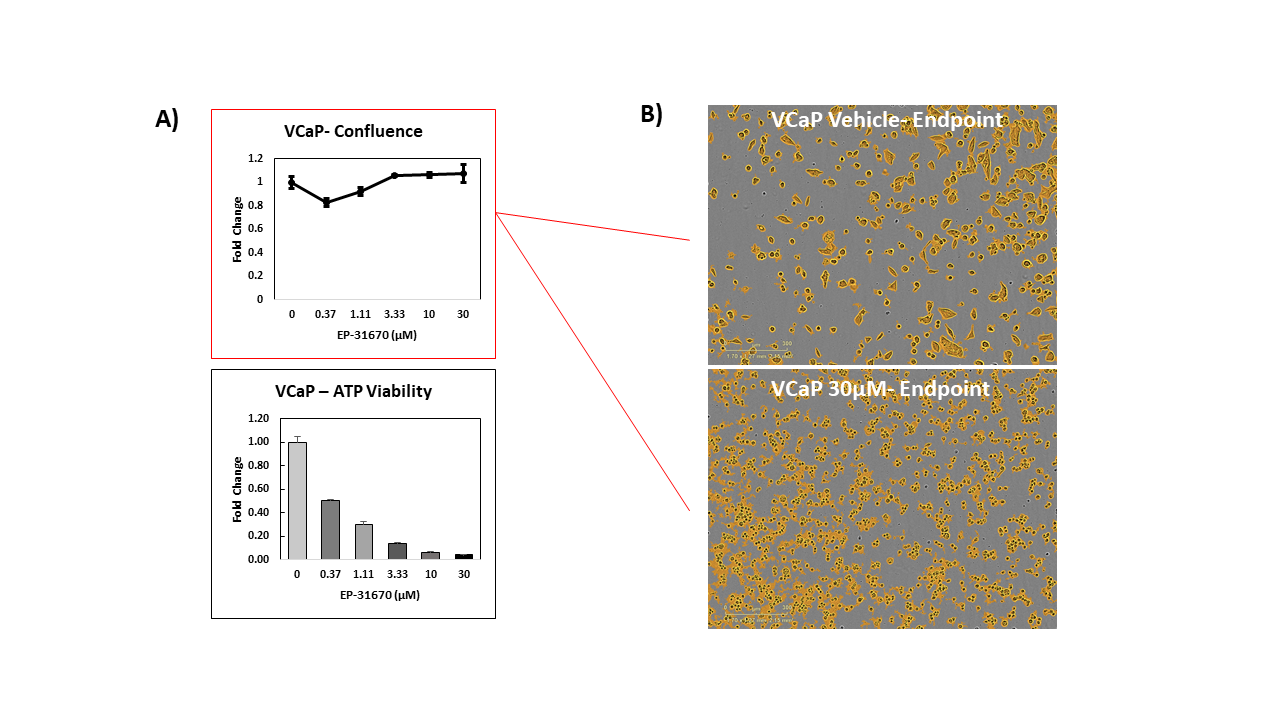

Supplement: S2 Fig — This figure was generated using data from the same experiment described in Fig 6. ADPC VCaP cells were seeded in a 96-well plate at optimized conditions and treated for 72-hours (as described in Fig 6). VCaP cell response to EP-31670 was estimated via A) endpoint confluence with the IncuCyte ZOOM and endpoint viability using CellTiter-Glo ATP viability data at 72-hrs for the tested cell lines. B) Comparing representative images from VCaP cells treated with vehicle vs 30μM show that at higher concentrations live-cell imaging can erroneously interpret dying/floating cells as viable cells (yellow outline). (TIF) [file pone.0287126.s004.tif]

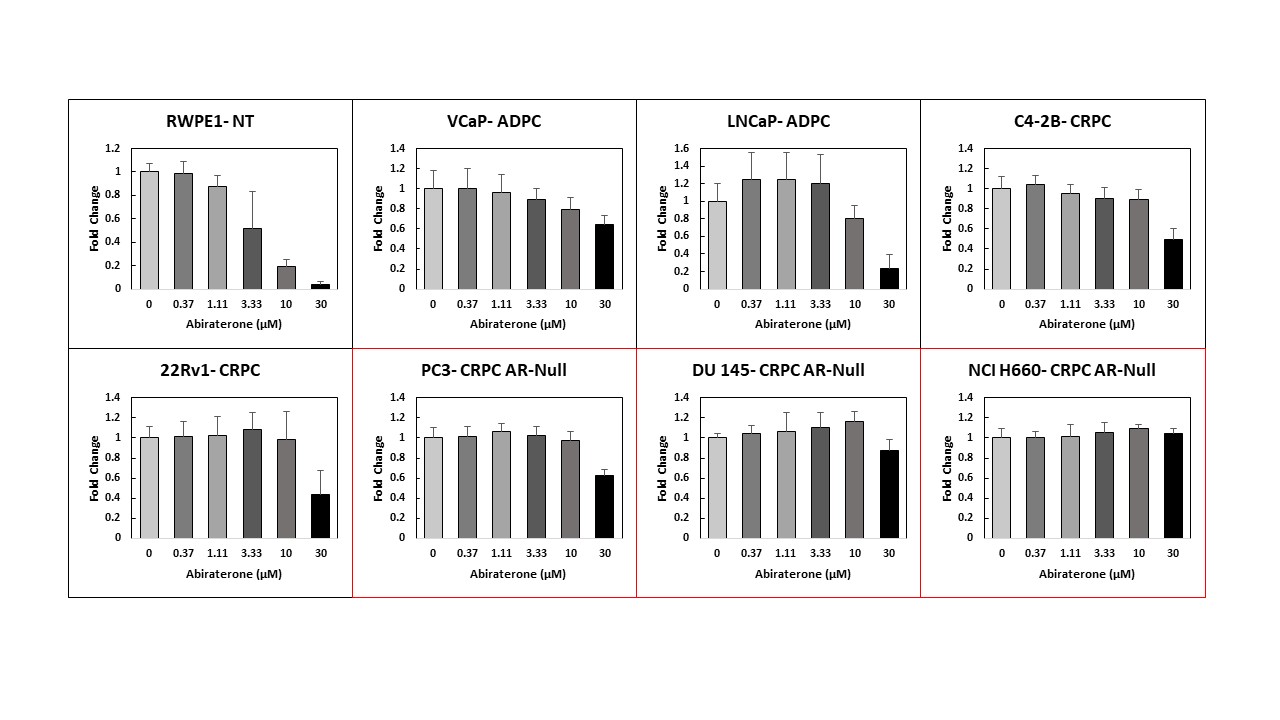

Supplement: S3 Fig — A panel of human prostate and cancer cells were seeded in a 96-well plate at optimized conditions (described in table 1) via the OT-2 liquid handler. After the cells adhered to the 96-well plate overnight, the AR antagonist abiraterone acetate was diluted via the OT-2 liquid handler to 0 (DMSO vehicle), 370nM, 1.11μM, 3.33 μM, 10 μM, and 30 μM (replicates of four wells per concentration) and cells were treated. Endpoint CellTiter-glo cell viability assay was completed. Dose-response curves for non-tumorigenic (NT) prostate cells RWPE-1, androgen-dependent prostate cancer (ADPC), LNCaP and VCaP, castration-resistant prostate cancer (CRPC) C4-2B and 22Rv1, androgen receptor (AR-null CRPC) DU 145 and PC3, and neuroendocrine prostate cancer (NEPC) NCI-H660 based on CellTiter-glo endpoint viability for at 72-hrs. Fold change growth normalized to the lowest drug concentration 0 (vehicle) for each concentration was calculated. ± SD (quadruplicate replicates). (TIF) [file pone.0287126.s005.tif]

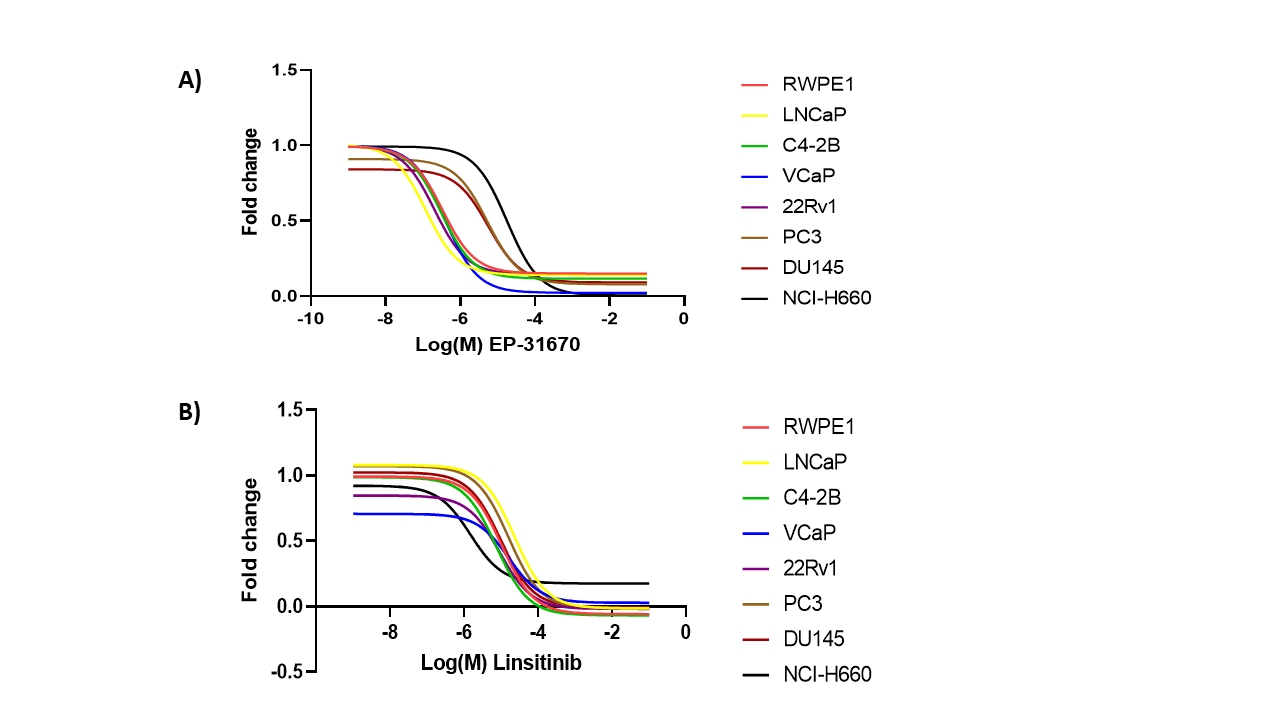

Supplement: S4 Fig — A panel of human prostate and cancer cells were seeded at optimized conditions and treated for 72-hours (as described in Figs 6 and 7) with A) the first-in-class BET and CBP/p300 dual inhibitor EP-31670 or B) the IGF-1R inhibitor linsitinib, respectively. For visualization, dose response curves were estimated using CellTiter-Glo endpoint viability at 72-hrs. Cellular drug response data were fit into a non-linear sigmoidal regression curve via and plotted together. Since the protocol screens only 5 drug concentrations, an algorithm to estimate these curves assigned values of Log(M) = -9 and Log(M) = -1 respectively to represent the two extremes of the curves (completely ineffective concentration and completely effective concentration). (TIF) [file pone.0287126.s006.tif]
