# Supplementary material for: Automation, live-cell imaging, and endpoint cell viability for prostate cancer drug screens
Source: PLoS One. 2023 Oct 10;18(10):e0287126. doi: 10.1371/journal.pone.0287126 (PMC10564233; doi:10.1371/journal.pone.0287126)
Supplement: Attachment — Submitted filename: Response to Reviewers F.docx [file pone.0287126.s007.docx]

PONE-D-23-06181

Automation, live-cell imaging, and endpoint cell viability for prostate cancer drug screens

PLOS ONE

**Overview of manuscript changes**

We thank the Academic Editor and reviewers for their insightful comments and suggestions, which have significantly improved our manuscript. The revised manuscript is a more detailed and accurate representation of the cell-line specific optimization and integration of live-cell imaging and endpoint viability approaches as requested by the Academic Editor and reviewers. The revisions better position this protocol as a useful tool for effectively screening new and repurposed drugs to identify effective compounds against advanced prostate cancer. In response to reviewer comments, the revised manuscript also includes new data (expanded Figure 7 (Fig 7) and four new supplemental figures (S2, S3, S4, and S5)). Lastly, as requested by the Academic Editor, we have made our protocol available through protocol.io: [dx.doi.org/10.17504/protocols.io.x54v9dojzg3e/v1](https://dx.doi.org/10.17504/protocols.io.x54v9dojzg3e/v1).

**Reviewer #1 (Comments to the Author (bold) and Response(s)):**

1. **All data presented are from end point assay (cell titer glo), which is just a manual method and has nothing to do with semi automation. It is unclear how the end point assay in this method differs from traditional end point assay. No data interpretation or results from imaging have been provided in detail. May be a correlation between cell titer glo and imaging-based data analysis be helpful to determine the consistency between the methods.**

We thank the reviewer for this important recommendation to better explain how cell-specific growth dynamics tracked via the IncuCyte ZOOM live cell analyzer and drug response as measured by the endpoint CellTiter-Glo viability assay were integrated to produce the summary tables in figures 6 and 7 (Fig 6D and Fig 7B). We have added the following text to the revised manuscript:

“In addition to representing drug efficacy as a direct function of changes in cell growth (compared to vehicle) across a drug concentration range, our approach also employs growth rate inhibition (GR) to produce drug response metrics. GR curves uniquely utilize cell growth dynamics to represent drug response. One method to calculate the values used to generate GR curves requires a time-dependent component such as the growth rate of cells in experimental conditions and a quantification of cellular response to tested drugs (i.e., endpoint confluence or cell metabolism). Our protocol used the IncuCyte ZOOM live-cell analyzer to determine vehicle treated cell line specific growth rates through 72-hrs in optimized conditions. We then used the measures of cell metabolism across the tested concentration range (vehicle-30uM) from the CellTiter-Glo endpoint viability assay to represent cell-specific drug response. For each experiment, the growth rates and drug responses for each cell line were assessed and integrated together to serve as the input values for the online GR calculator Tool provided by the Sorger Lab at Harvard Medical School … In most cases drug response as assessed by endpoint confluence produced similar results to CellTiter-Glo and sufficed as input data for GR curves (supplemental figure S2). However, by assessing drug response through ATP-based cell viability we avoid possible false negative classifications which can occur when imaging platforms erroneously include dead/floating cells in confluence assessment (supplemental figure S3).”

2. **AR antagonist screen will be helpful to determine if the method differentiates between AR-positive and -negative cells in terms of growth inhibitory effect.**

We thank the reviewer for this suggestion and have added a new supplemental figure S4 which reports the screening results of Abiraterone Acetate. We have added the following text to the revised manuscript:

“We also demonstrate differential drug efficacy for the CYP17 inhibitor / AR antagonist abiraterone acetate but only at the 10µM and 30µM concentrations for the AR-expressing non-tumorigenic cell line (RWPE-1) and the AR-expressing (especially LNCaP and VCaP) compared to the AR-null PC cell lines (PC3, DU 145, and NCI-H660) (supplemental figure S4). AR signaling inhibitors (ARSIs) including abiraterone acetate are typically used in the ≥ 10µM range and cause mostly cytostatic effects (cell death is less pervasive). Furthermore, ARSIs typically require longer times (more than 72 hrs) to see effects on cell growth. For these reasons, ARSIs as single compounds do not produce robust cell growth inhibitory effects with this platform.”

**Reviewer #2 (Comments to the Author (bold) and Response(s)):**

1. **It is not clear how “optimal assay conditions” are defined. Is it the linear relationship between cell metabolism and seeding density, as shown and implied in F4? That ignores the dynamic range of the assay to measure growth effects for drug screening. The abstract claims to have demonstrated reliability and reproducibility in the reported screening approach, however, no measurements of reliability or repeatability are presented. These latter quantifiable metrics would be strong measures to define optimal conditions.**

We apologize for not explaining this point with sufficient clarity and have revised the narrative as follows:

“As our system integrated both live-cell imaging and endpoint viability, a critical step of our protocol was to specifically optimize for sequential use of both approaches. The general requisites for optimization were that endpoint cell density must remain within the detectable linear range of the CellTiter-Glo assay, and that the IncuCyte ZOOM could verify continued proliferation of the control cells without achieving over-confluence within 72-hrs…”

“At the 72-hr time point, cells were analyzed with the ATP viability CellTiter-Glo assay. Since cells were seeded in equal increments, we expected a linear relationship between ATP synthesis (raw luminescence values) and cell seeding densities. Thus, the first requirement of optimization was the determination of seeding densities and culture conditions that allowed for cell confluence after 72 hrs to be within the detectable linear range of the CellTiter-Glo ATP viability assay. Additionally, we used analyses of the live-cell imaging data to determine the cell-specific growth dynamics to ensure that cells did not exceed ~70% confluence. Another requirement was that the vehicle treated cells continued to proliferate through the duration of the experiment.”

Regarding reliability and reproducibility, in the abstract we were referring to the assay’s ability to reproduce other lab findings which previously defined drug responses for select cell lines such as the first-in-class BET and CBP/p300 dual inhibitor EP-31670 with ADPC and CRPC cell lines (PC3 and DU 145), and with our previously published findings with an IGF1-R inhibitor in ADPC VCaP cells. We have updated the abstract to read as follows:

“We demonstrated effectiveness and reliability of this pipeline through validation of the established finding that the first-in-class BET and CBP/p300 dual inhibitor EP-31670 is an effective compound in reducing ADPC and CRPC cell growth.”

2. **The cell numbers plated and growth conditions lead to low confluence at the end of 72hrs. The advantages of this over higher confluence at 72 hrs (the premise from F3A) is unclear.**

We thank the reviewer for raising this point and the opportunity to better explain why it is important to have endpoint confluence lower than ~90% as demonstrated in figure 3 (Fig 3A). We have added the following text to the revised manuscript:

“A limitation of live-cell imaging to evaluate cell growth and drug efficacy is that cell confluence/density estimations become less accurate with higher confluence. This is due to the propensity of cells to clump (i.e. grow into the vertical plane as they proliferate). Furthermore, at higher endpoint confluence, cells treated with effective compounds tend to detach and this can obstruct visualization of adherent cells. These issues make it difficult to accurately capture representative images of attached cells, assess confluence, and measure drug efficacy.”

3. **Was there cell loss with removing media following 24hrs of plating. Experience of many screening labs indicates that removing media from 96 well plates can lead to loss of cells.**

We thank the reviewer for this important question. Our protocol was designed to avoid cell loss as typically experienced by other screening labs. We have added the following text to the revised manuscript:

“During the development of the Opentrons OT2 protocol, we determined that removing/adding media to cells at a flow rate of 5 µL/sec with the pipette tip positioned ~1mm off center prevented significant cell loss/detachment.”

4. **“Real-time imaging demonstrated EP-31670 efficacy in prostate cancer as CRPC C4-2B cell proliferation was reduced even in the sub-nanomolar range (Fig 6A).“ The authors must mean sub-micromolar range, which is what the data show.**

Thank you for identifying this error. The manuscript has been corrected and the phrase has been changed to “sub-micromolar range”.

5. **F6C and F7A-D data should be plotted as dose response curves not a bar graph.**

We understand the reviewer’s request and now include estimated dose response curves of the data as supplemental figure S5. However, we respectfully request that the reviewer accepts the representation of the data as histograms for the primary figures as we believe that it is easier to compare differences in drug response across cell lines. Dose response curves are superior to histograms when there are more than five drug concentrations being tested so that a complete and accurate curve can be fit. We also want to point out that we include IC50 values and AUC values in a summary table beneath the histograms which account for any “at-a-glance” benefits that traditional sigmoidal dose-response curves may hold.

6. **There is no integration of the two (three if you count morphology) endpoints of the approach. Is final cell number (implied from a metabolic assay) or the determined growth rate (from imaged cell confluence) advantageous over the other for detecting “hits” or is using the two provide more sensitivity or selectivity for “hits”. The authors reference a manuscript in preparation of a screen of over 500 compounds, so this manuscript seems to be a companion methods paper. The challenge is that the screen data in that manuscript in preparation may be important to understand any advantages of the approach reported here.**

We thank the reviewer for this important recommendation to better explain how cell-specific growth dynamics tracked via the IncuCyte ZOOM live cell analyzer and drug response as measured by the endpoint CellTiter-Glo viability assay are integrated to produce the summary tables in figures 6 and 7 (Fig 6D and Fig 7B). We have added the following text to the revised manuscript:

“In addition to representing drug efficacy as a direct function of changes in cell growth (compared to vehicle) across a drug concentration range, our approach also employs growth rate inhibition (GR) to produce drug response metrics. GR curves uniquely utilize cell growth dynamics to represent drug response. One method to calculate the values used to generate GR curves requires a time-dependent component such as the growth rate of cells in experimental conditions and a quantification of cellular response to tested drugs (i.e., endpoint confluence or cell metabolism). Our protocol used the IncuCyte ZOOM live-cell analyzer to determine vehicle treated cell line specific growth rates through 72-hrs in optimized conditions. We then used the measures of cell metabolism across the tested concentration range (vehicle-30uM) from the CellTiter-Glo endpoint viability assay to represent cell-specific drug response. For each experiment, the growth rates and drug responses for each cell line were assessed and integrated together to serve as the input values for the online GR calculator Tool provided by the Sorger Lab at Harvard Medical School … In most cases drug response as assessed by endpoint confluence produced similar results to CellTiter-Glo (supplemental figure S2) and sufficed as input data for GR curves. However, by assessing drug response through ATP-based cell viability we avoid possible false negative classifications which can occur when imaging platforms erroneously include dead/floating cells in confluence assessment (supplemental figure S3).”

Regarding the advantages of using our integrated approach for the identification of “hits” we have added the following text to the revised manuscript:

“For each experimental run using this protocol, three key data types were obtained: 1) cell-specific drug response represented as endpoint cell viability and measured by the CellTiter-Glo assay. 2) Live-cell imaging via the IncuCyte ZOOM platform which quantifiably tracks real-time confluence, cell growth dynamics, and permits qualitative assessments of morphological changes as a response to drug treatment. 3) A suite of data including drug efficacy metrics IC50, AUC, GR50, and AOC which were calculated through the integration of both cellular growth rates as determined through the IncuCyte ZOOM imaging platform and the endpoint cell metabolism ATP viability assay data. These drug screens provide all necessary data to draw conclusions about drug efficacy across a heterogeneous panel of cell lines while avoiding the shortcomings of each individual approach including potential false negative classification as discussed above.”

7. **Only single drug effects are reported. In cancer therapy, drug combinations are clearly needed. What advantages do the combined readouts provide for drug combination screens?**

We appreciate the reviewer’s question regarding the advantages that our integrated screening method would provide to drug combination screens. Indeed, we envision adapting our protocols for this purpose, but of course this will require optimization and the development of an additional arm of the protocol. We acknowledge this in the following quote from the discussion section of the revised manuscript:

“We designed and optimized this platform to screen single compounds with a future goal of expanding this type of analysis to drug combinations.”

We predict that the advantages the combined readouts provide for combination screens will be akin to those experienced through the utilization of this platform for single drug screens: “integration of live-cell imaging and endpoint viability assays: providing visual representation of overall cell viability and integrity throughout experiments, endpoint drug performance metrics, and identification of cytostatic vs cytotoxic effects of tested drugs.” This platform will permit the capture of the effectiveness of the combined drug response at various concentrations but will also capture the comparative morphological changes which occur because of combined therapy which could grant preliminary insight to potential mechanisms of action.

**Reviewer #3 (Comments to the Author (bold) and Response(s)):**

1. **Line 170 and 171 say that "BPH-1 cells cultured in media supplemented with 2% FBS and seeded at 500 cells/well represented the optimal assay conditions". But in the figure 4, BPH-1 cells are shown to have cell seeding density at 200 cells/well. PLease explain this confusing numbers.**

We apologize for the confusion. The correct seeding density for BPH-1 cells in our 96-well plate assay is 500 cells/well as listed in Table 1. Figure 4 (Fig 4) represents the seeding density range that was tested during optimization to verify that the tested seeding densities would be within the detectable linear range of the endpoint ATP viability assay. This range includes 250 cells/well, 500 cells/well, 750 cells/well, and 1000 cells/well. The X-axis began at 200 cells/well and increased at increments of 200 cells/well to 1000 cells/well, this labeling was misleading and has since been updated to begin at 250 cells/well and increase in increments of 250 cells/well to 1000 cells/well.

2. **Also, the authors did not clearly explain the use of AUC and AOC in their assays. They need to explain what were these calculations used to evaluate in terms of cell viability or growth or inhibition.**

We thank the reviewer for this important recommendation to better explain how the quantitative metrics represented in figure 6 (Fig 6D) and figure 7 (Fig 7B) area under the curve (AUC) and area over the curve (AOC) are derived and the value that they add to data analysis and interpretation in our system. The following text has been added to the revised manuscript:

“The metrics AUC (area under the curve) and AOC (area over the curve) are calculations of the area beneath the traditional sigmoidal dose response curves or the area above the GR dose response curve. We incorporated AUC and AOC values because although IC50 and GR50 are the standard for drug efficacy data, they have a critical limitation when used in dose-limited comparative screening efforts. If the tested drugs do not inhibit cell growth sufficiently within the tested drug concentration range, then these values cannot be calculated. AUC and AOC values are derived from the integral of the sigmoidal dose response curve (AUC) or GR curve (AOC) from the lowest tested concentration (0 in our screen) concentration to the max concentration (30µM in our screen). In the case of AUC, a completely ineffective compound at every concentration will have a linear curve and maintain a fold change value of 1 and have a higher AUC. Conversely, a compound that is effective at the lowest tested concentrations will have a lower AUC. The closer to 0 the AUC the more effective the compound is. When evaluating GR curves, in which x-axes range from -1 through 1, the most effective compounds will have values closer to -1 at cytotoxic concentrations and ineffective compounds will be closer to 1. Since GR curves calculate area over the curve, the most effective compounds will have a larger AOC value. When evaluating compound efficacy in cases where IC50 or GR50 cannot be calculated, comparing the AUC and AOC values permits quantifiable assessments across cell lines.”

**Reviewer #4 (Comments to the Author (bold) and Response(s)):**

1. **While not particularly novel, this is a good protocol paper for drug screens in prostate cell lines - which are notoriously difficult to work with. It has merit for publication, despite the limited amount of screen data presented. These data regarding the example drugs would be nice to include for all cell lines.**

We appreciate the reviewer’s suggestion. We have now updated Figure 7 (Fig 7) and included data from all tested cell lines. The newly added supplemental figure S4 also includes all tested cell lines.
